# Ethical by Design: Engaging the Community to Co-design a Digital Health Ecosystem to Improve Overdose Prevention Efforts Among Highly Vulnerable People Who Use Drugs

**DOI:** 10.3389/fdgth.2022.880849

**Published:** 2022-05-26

**Authors:** Kasey R. Claborn, Suzannah Creech, Quanisha Whittfield, Ruben Parra-Cardona, Andrea Daugherty, Justin Benzer

**Affiliations:** ^1^Steve Hicks School of Social Work, The University of Texas at Austin, Austin, TX, United States; ^2^Department of Psychiatry, Dell Medical School, The University of Texas at Austin, Austin, TX, United States; ^3^Addiction Research Institute, The University of Texas at Austin, Austin, TX, United States

**Keywords:** overdose prevention, community engaged research, surveillance, human factor, harm reduction

## Abstract

**Introduction:**

The COVID-19 pandemic highlighted significant structural barriers that exacerbated health inequities among people at-risk for overdose. Digital health technologies have the potential to overcome some of these barriers; however, development of these technologies often fails to include people who use drugs and community key stakeholders in the development and dissemination process. Consequently, this may exacerbate health inequities and the digital divide among underserved, highly vulnerable people who use drugs.

**Methods:**

The current study employed community-engaged research methods to develop and implement a digital platform to improve overdose surveillance among harm reductionists in Texas. We used a co-design process with four community advisory boards (CABs) and conducted qualitative interviews among *N* = 74 key stakeholders (*n* = 24 people who use drugs; *n* = 20 first responders, *n* = 20 harm reductionists, *n* = 10 overdose prevention and response experts) to inform initial design and development.

**Results:**

Several key themes emerged through the qualitative data pertaining to technical features and human factors applications. In regards to technical features, participants highlighted the importance of developing a unified system of overdose reporting and data sharing among community organizations within a county or region to better inform overdose surveillance and community outreach efforts. This system should include flexible data entry methods, have offline usage capability, be user friendly, and allow for tracking of overdose-related supply distribution. Key human factor themes included the need to use person-centered language, to preserve the established trust of the community organizations among people who use drugs, to be tailored to specific target user groups (e.g., harm reduction workers, people who use drugs, first responders), and maintain transparency of data usage. Further, participants noted the importance of developing a platform that will facilitate client conversations about overdose when doing outreach in the field. These themes were reviewed by our CABs, academic, and industry partners to design an overdose digital platform uniquely tailored to community-based organizations providing harm reduction and overdose response efforts.

**Discussion:**

Community engagement throughout the development process is critical toward developing digital health tools for underserved people who use drugs. Dismantling the power structure among academic and industry partners is critical toward creating equity in engagement of community-based partners, particularly among persons with lived experience in addiction, a history of incarceration, or financial challenges. Our study highlights a multisectoral co-design process across community-academic-industry partners to develop a digital health tool tailored to the unique needs of community-based harm reduction organizations serving highly vulnerable people who use drugs. These partnerships are essential toward creating impact and reducing health disparities among highly vulnerable people who use drugs.

## Introduction

Overdoses involving opioids and other substances were declared a public health emergency in the United States in 2017 and have reached historically devastating numbers during 2021 (1). Recent data from the CDC (2) indicated that over 108,000 Americans died as a result of drug overdose during 2021, an increase of over 30% from previous years (3). It is critically important to highlight that fatal and non-fatal overdose data are likely severely underreported in the United States due to insufficient surveillance methods and systemic gaps in overdose data (2). Existing overdose estimates rely almost exclusively on data from emergency management systems (EMS), emergency departments (ED), and death records, reflecting only PWUDs who interact with the healthcare system following overdose (4). Current overdose data collection methods are fragmented, insufficient, and act to marginalize people who use drugs (PWUD) in analyses (1, 5).

These data are often housed in disparate systems which limit opportunity for integration and systematic analysis (6). Importantly, many individuals who experience an overdose do not contact the emergency management system (EMS) or interact with the healthcare system due to stigma and fear of legal repercussions. Existing overdose data sources rely heavily on data from EMS, emergency departments, and death records to calculate public health statistics. Consequently, only individuals who encounter the health care system following an overdose are recorded within these statistics. Capturing overdose data among hidden populations who do not access these systems is critical for a comprehensive and equitable strategic overdose response.

Different approaches need to be considered in digital health technology development targeting PWUD, particularly among doubly vulnerable minority populations. Employing community engaged research methods through co-collaboration with PWUD throughout technology design and development works to mitigate exclusion of the very population it is meant to serve (7). When integrating ethical considerations during the planning phase, digital health platforms can become “ethical by design” (7). Integrating the needs and voices of PWUD through community engagement and collaboration during the planning, implementation, and dissemination of a digital health platform is necessary to take “ethical by design” one step further to become “equitable by design.”

Community based participatory research (CBPR) is a co-collaborative model that re-aligns traditional “researcher-subject” hierarchies to promote partnership and respond to community priorities (8). The community through CBPR becomes a part of the research team (8). Central to health equity-oriented approaches is the inclusion of PWUD throughout planning, development, and implementation (1). Community involvement increases trust and efficacy of the resulting product within vulnerable populations (7). CBPR provides a trajectory to remedy historical racial/ethnic and socioeconomic inequities through co-collaboration with marginalized groups (8). Encompassing strategies such as community coalitions (8), qualitative interviews, and leading with a “nothing about us without us” (9) perspective, marginalized communities become co-collaborators and integral contributors to digital health and other solutions aimed at their community. Performing community level engaged research improves implementation, addresses stigma, and acts to improve the analysis and understanding of the data by providing additional context.

Texans Connecting Overdose Prevention Efforts (TxCOPE) is a digital health ecosystem developed through employment of CBPR, a community engaged research approach. Similar to other states in the United States, Texas currently does not have a unified, comprehensive digital system in place for fatal and non-fatal overdose reporting and tracking, contributing to the gap in comprehensive, real-time collection, dissemination, and analysis of overdose data (1). TxCOPE will have four interconnected platforms with each tailored to fit the needs of harm reduction organizations, the general community, first responders, and healthcare providers. TxCOPE was conceptualized and is being designed with equity in mind through the utilization of community advisory boards (CABs) made up of various stakeholders with representation from people with lived experience, harm reduction, prevention, and treatment organizations throughout pilot counties, and use of qualitative interviews with PWUD, first responders, and harm reduction organizations. The CABs, in both urban and rural communities within Texas, worked to identify local challenges (5) facing PWUD while informing the design of this digital health platform. This manuscript describes key findings from our CBPR approach to develop and implement a digital platform to improve overdose surveillance and community prevention and response efforts in Texas.

## Methods

### Theoretical Framework and Methodological Approach for Co-design Process

**Figure 1:**
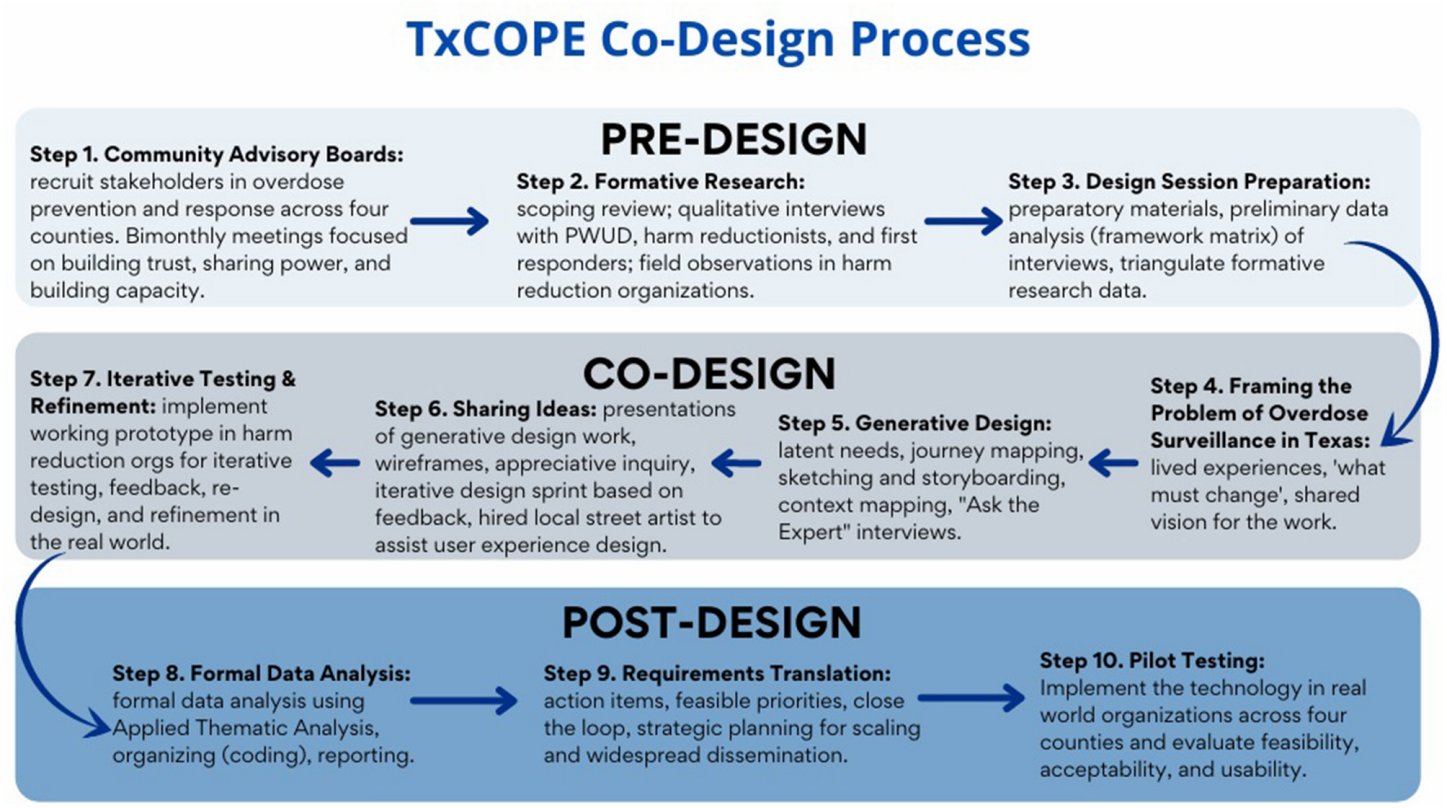
Community-Academic technology co-design process.

We employed principles from community-engaged research and user-centered design to inform our co-design process and formative research approach. Community-engaged research re-aligns typical researcher-subject hierarchies to involve communities, elevate their perspective within the research and solutioning process, and dismantle existing power structures (8, 10). User-centered design is an iterative process that engages a multidisciplinary team based on the active involvement of end users to improve understanding of the user and task requirements throughout the design and development process (11). Co-Design marries these approaches and extends the role of end users. Co-Design is a method for designing digital health technologies with, not for, the target user group that focuses on mutual learning, trust, shared decision-making, and open and active communication (12). Our Co-Design process included people with lived experience (PWUD), the community (harm reduction and overdose prevention stakeholders), academic researchers, and technologists working together to improve overdose reporting and surveillance methods in Texas. Our target user groups for the TxCOPE platform were harm reduction organizations and PWUD. McKercher (12) outlined four key principles for co-design: (a) share power–acknowledge and address power differentials associated with decision-making, design, delivery, and evaluation; (b) prioritize relationships–establish trust among co-designers, funders, and organizers and build a strong social connection prior to co-design; (c) use participatory means–use design methods that facilitate discovery and move people from participants to active partners; and (d) build capacity–researchers take the role of coach instead of “expert” to facilitate shared understanding and develop champions of the technology to support real world implementation and sustainability. We engaged a multisectoral group of partners across the community, academic, and technology sectors to create an immersive, highly creative environment for health innovation focused on solving the problem of needing real-time, reliable overdose data surveillance. Our approach closely modeled the recently published framework of Bird and colleagues (13) which outlined the Generative Co-Design Framework for Healthcare Innovation (see Figure 1).

### Pre-design

Our pre-design process consisted of three steps: establishing community advisory boards, conducting formative research, and preparing for the co-design sessions.

### Community Advisory Boards

One method in community-engaged research is the development of community coalitions of key stakeholders and individuals with lived experience (5). In this study we established Community Advisory Boards (CABs) across four pilot counties spanning diverse cultures and urban and rural settings in Texas. CAB members were composed primarily of local harm reduction organization leaders and other representatives of community agencies active in the field such as first responders, treatment, and prevention providers. To recruit CAB members, we first identified relevant community-based organizations in each county across the following sectors: harm reduction, substance use prevention and/or treatment, first responders (EMS/Fire), and medical examiner office. We contacted leadership in each organization and described the project and the role of the community advisory board in the co-design of the overdose reporting platform. Organizations who agreed to have representation then identified a champion within their organization to serve as a CAB member. Each CAB met every 2 months for 60–90 min over a period of 2 years. CAB activities are outlined in Table 1.

**Table 1:** Summary of co-design activities and outputs.

**Step**	**Co-design activities**	**Outcomes and outputs**
**Pre-design**
1: Community advisory boards	• Recruit CAB members • Establish community-based partnerships and operational structure • Bimonthly CAB meetings	• Multisectoral partnerships across four community advisory boards • Build community trust, social connections, and capacity for design & implementation
2: Formative research	• Scoping review of existing overdose surveillance methods and data dashboards • Qualitative interviews • Field observations	• Define existing technologies • Describe existing data dashboards • Data from people with lived experience • Understand existing organizational work flow and barriers/facilitators to overdose reporting in organizations
3: Design session preparation	• Preliminary data analysis • Triangulate data from Step 2 • Training project team in co-design process	• Materials for Design Session workshops
**Co-design**
4: Framing the problem	• *Design Session 1:* presenting formative research data to CABs for community data analysis and interpretation • Brainstorming and solutioning • Assess capacity of harm reduction partners to co-create the technology • Assess how community defines success	• In-depth understanding of the problem • Understanding shared vision for technology design and implementation • Establish community-defined success metrics
5: Generative design	• *Design Session 2:* generative design work • Journey mapping • Sketching and storyboarding • Context mapping • “Ask the Expert” interviews	• Define the target user experience • Identify steps in the user's journey • Generate preliminary features and core components of the technology • Design team uses outputs to develop wireframes
6: Sharing ideas	• *Design Session 3:* present generative design work and wireframes • Inquiry and feedback	• Low-fidelity prototype
7: Iterative testing and refinement	• Implement working prototype in harm reduction organizations • Iterative feedback and revision	• Final product design and development of high-fidelity prototype
**Post-design**
8: Formal data analysis	• Applied thematic analysis of qualitative interviews	• Reports and presentations of process and outcome evaluation findings
9: Requirements and translation	• Strategic planning for scaling and widespread dissemination	• Dissemination and implementation protocol
10: Pilot testing	• Implement and evaluate in real world	• Data on feasibility and acceptability

### Formative Research

Activities during this step included conducting a scoping review, series of field observations among harm reduction organizations and street outreach teams, and qualitative interviews with key stakeholders. These data were triangulated to understand existing overdose surveillance methods, gaps in data collection and reporting, and perceived solutions to improve overdose data to inform community response efforts. Qualitative interviews were conducted among a series of *N* = 74 key stakeholders and people with lived experience (*n* = 24 people who use drugs; *n* = 20 first responders, *n* = 20 harm reductionists, *n* = 10 overdose prevention and response experts) across the four pilot counties in Texas to inform initial design and development. These interviews were between 60 and 90 min in duration and participants received compensation in the amount of $30 for their time. The following research questions were addressed in interview: (1) How do stakeholders engage in the field of drug overdose perceive existing overdose and naloxone data in the State of Texas?; (2) How do stakeholders engaged in the field of drug overdose currently report opioid-related data? (overdose info, naloxone administration/distribution, etc.); (3) What are the perceived problems or negative outcomes associated with underreporting overdoses in Texas?; (4) What are stakeholders engaged in the field of drug overdose perspectives on other existing data reporting programs?; (5) What are perceived solutions to improve the tracking of overdose-related variables?; and (6) What methods should be used to implement this system to promote widespread adoption and sustainability?

In interviews with PWUD, the research questions addressed varied slightly and reflected the following: (1) Do people who use drugs currently report overdose-related data? If yes, among this population who is more likely to report and why? If no, what are the reasons this population does not report and how often do they think overdoses occur and are not reported?; (2) What methods will increase the likelihood that people who use drugs will report overdose-related data (e.g., what would incentivize reporting behavior)?; and (3) What methods should be used to increase adoption and sustainability of an overdose reporting system among people who use drugs? Semi-structured interview guides and debriefing guides were created for each interview group. The interview guides were composed of structured, open-ended question, and provided flexibility for the interviewers to adapt and clarify questions as needed. Methods and results for the qualitative interviews are described below.

### Co-design Phase

During the Co-Design Phase, our academic team lead coordination of all aspects of the project and facilitated meetings with our technology team, Maven Wave an Atos company, and our community partners. This included multisectoral meetings focused on framing the problem, conducting generative design work, sharing ideas for technology design and development, reviewing wireframes and low-fidelity prototypes, and iterative testing and refinement. We completed preliminary data analysis throughout this phase using the framework matrix method for efficiency.

### Post-design Process

The Post-Design Phase consisted of a pilot test across participating harm reduction organizations to assess feasibility and refine the technology. Data were formally analyzed during this phase using Applied Thematic Analysis. Finally, we developed a plan for scaling and implementation. This included hiring a local street artist to develop promotional materials and artwork. Table 1 outlines activities and outputs for all phases of the co-design process.

### Participants

#### Eligibility

The inclusion criteria for qualitative interviews with medical examiners, justices of the peace, harm reductionists, and other key stakeholder representatives included: (1) eighteen years or older, (2) employed in one of the target counties (e.g., medical examiner, justice of the peace, etc.) or have relevant experience that will inform statewide efforts; and (3) ability to read and speak in English. The inclusion criteria for people who use drugs (PWUD) included: (1) eighteen years or older, (2) reported misuse of opioids or stimulants in the past 3 months, (3) resides in Texas, and (4) ability to read and speak in English and/or Spanish. The exclusion criteria for all participants included: the inability or unwillingness to provide consent, being actively suicidal, or psychotic.

#### Recruitment

Screening for prospective participants consisted of a short (5–10 min) screening survey conducted over the phone or through email and was coordinated by the research team. When the inclusion criteria were met, the research team would then provide and obtain informed consent. During the consent process, participants were informed about the purpose of the study and all procedures. Participants were told that the interview would be audio-recorded and then transcribed verbatim. The transcripts would be cleaned and all identifying information would be removed then audio-recordings would be deleted. Participants were given time to review the consent form in-depth and ask questions. Participants were given a copy of the consent form for their records. Participants protected health information were removed and had a unique ID number assigned to them. Recruitment methods were comprised of in-person (when permitted), flyers, e-mails, telephone, snowball sampling, social media advertising, web-posting, word of mouth, and using CABs in each pilot county to assist with recruiting. In total, we recruited and interviewed 24 people who use drugs, 20 harm reductionist, 20 first responders, and 10 overdose prevention and response experts before reaching data saturation.

### Data Collection

Qualitative interviews were conducted by videoconference and in-person, when permitted, with two trained researcher staff. One researcher conducted and led the interview while the other co-facilitated and took notes. After the interview was completed, the audio recordings were transcribed verbatim by a confidential professional transcription agency. Transcripts were then cleaned and scrubbed of all personal identifying information. Once returned, the cleaned transcripts and the debriefing guides were used for analysis.

### Data Analysis

Qualitative interview data were analyzed using applied thematic analysis and triangulated to inform development of the TxCOPE digital ecosystem. Data from the qualitative interviews were analyzed using applied thematic analysis which was selected for its flexibility and systematic approach in analyzing text-based qualitative data while planning as well as preparing for the data collection (14). The research team identified emergent themes based on the a priori research goals. These major themes informed the development of working codebooks and framework matrices for the respective interviewee (first responder, harm reductionist, pwud, general stakeholder). The general stakeholder codebook outlined the following data: overdose, current overdose reporting, non-traditional first responders, solutions to improve reporting, digital platform structure, and digital platform implementation. The PWUD codebook outlined the aforementioned data with the addition of perceptions of organizations. The harm reductionist codebook outlined the data included in general stakeholder codebook with the addition of the following: marketing and branding and stigma.

Data analysis was conducted by six trained coders (two clinical research associates and four research assistants) using a reflexive analysis approach. The process involved assigning two team members per transcript to be coded independently using the corresponding codebook. Once independent coding was completed the two coders met to resolve discrepancies in the coding using the reflexive team approach to resolve any discrepancies. Once a consensus was met the coders finalized the coded transcript. During coding additional relevant data and themes emerged resulting in multiple revisions of the codebook. In order to organize the data collected during interviews, framework matrices were developed. The debriefing guides and cleaned transcripts were used to identify emerging themes and house direct quotations from the interviews. Once the data reached saturation, the emergent themes were collected from the coded transcripts and framework matrices. Data saturation was determined to be achieved when no new information was obtained in new interviews on key research questions (15). Several key themes in human factor (language use, trust, and transparency) and technical features (unified system, various data entry methods, and data sharing) emerged that will be discussed further within the results Section.

## Results

### Participants

Qualitative interviews with 74 key community stakeholders (*n* = 24 people who use drugs; *n* = 20 first responders, *n* = 20 harm reductionists, *n* = 10 overdose prevention and response experts) were conducted. Participants included: emergency department and hospital employees (11.1%), EMS (22.2%), epidemiologists (3.7%), fire department (12.9%), harm reductionist (35.1%), law enforcement (1.9%), poison control (1.9%), substance use treatment providers (5.6%) and other key stakeholders (state health department official, technical assistant and workforce development, mental health peer specialist) (5.6%). See Table 2 for Participant characteristics. Thematic analysis revealed important information regarding how the TxCOPE digital platform could be designed to meet the needs of these diverse stakeholders. Results are organized with regard to preservation of trust, preferences regarding content and digital features, and participants' perspectives of the opportunities and concerns regarding the digital platform.

**Table 2:** Participant characteristics (*N* = 74).

	**People who use drugs** **(*n* = 24)**	**First responders** **(*n* = 20)**	**Harm reductionists** **(*n* = 20)**	**Overdose prevention/ response experts** **(*n* = 10)**	**Total** **(*n* = 74)**
	***N (%)***	***N (%)***	***N (%)***		***N (%)***
**Age**					
18–24	4 (16.6)	1 (5.0)	1 (5.0)	0 (0.0)	6 (8.1)
25–34	4 (16.6)	6 (30.0)	8 (40.0)	3 (30.0)	21 (28.3)
35–44	11 (45.8)	6 (30.0)	5 (25.0)	3 (30.0)	25 (33.7)
45–54	2 (8.3)	6 (30.0)	3 (15.0)	3 (30.0)	14 (18.9)
55+	3 (12.5)	1 (5.0)	3 (15.0)	1 (10.0)	8 (10.8)
**Sex at birth**					
Male	13 (54.2)	18 (90.0)	10 (50.0)	1 (10.0)	42 (56.7)
Female	11 (45.8)	2 (10.0)	10 (50.0)	9 (90.0)	32 (43.2)
**Gender identity**					
Man	13 (54.2)	17 (85.0)	9 (45.0)	1 (10.0)	40 (54.0)
Woman	11 (45.8)	2 (10.0)	9 (45.0)	9 (90.0)	31 (41.9)
Genderqueer	0 (0.0)	1 (5.0)	2 (10.0)	0 (0.0)	3 (4.1)
**Race**					
African American or Black	1 (4.2)	0 (0.0)	2 (10.0)	0 (0.0)	3 (3.9)
Asian	0 (0)	2 (10.0)	2 (10.0)	2 (20.0)	6 (7.9)
White/ Caucasian	19 (79.2)	17 (85.0)	14 (70.0)	8 (80.0)	58 (76.3)
American Indian or Alaska Native	0 (0.0)	0 (0)	1 (5.0)	0 (0.0)	1 (1.3)
Native Hawaiian or Pacific Islander	0 (0)	0 (0)	0 (0.0)	0 (0.0)	0 (0.0)
Other	6 (23.0)	1 (5.0)	1 (5.0)	0 (0.0)	8 (10.5)
**Ethnicity**					
Hispanic or Latino	10 (41.7)	3 (15.0)	8 (40.0)	1 (10.0)	22 (29.7)
Non-hispanic or Latino	13 (54.2)	16 (80.0)	11 (55.0)	9 (90.0)	49 (66.2)
Other	1 (4.2)	1 (5.0)	1 (5.0)	0 (0.0)	3 (4.1)
**Religion**					
Christian	6 (25.0)	9 (45.0)	6 (28.6)	5 (50.0)	26 (34.6)
Buddhist	1 (4.2)	0 (0.0)	1 (4.8)	0 (0.0)	2 (2.6)
Jewish	1 (4.2)	0 (0.0)	0 (0.0)	0 (0.0)	1 (1.3)
Muslim	0 (0)	0 (0.0)	0 (0.0)	1 (10.0)	1 (1.3)
Atheist	2 (8.3)	6 (30.0)	6 (28.6)	2 (20.0)	16 (21.3)
Hindu	0 ()	0 (0.0)	0 (0.0)	1 (10.0)	1 (1.3)
Other	14 (58.3)	5 (25.0)	8 (38.0)	1 (10.0)	28 (37.3)
**Education level**					
Some grade school	1 (4.2)	0 (0.0)	0 (0.0)	0 (0.0)	1 (1.35)
Some high school	1 (4.2)	0 (0.0)	0 (0.0)	0 (0.0)	1 (1.35)
High school diploma or GED	8 (33.3)	0 (0.0)	2 (10.0)	0 (0.0)	10 (13.5)
Some college or 2-year degree	12 (50)	10 (50.0)	3 (15.0)	1 (10.0)	26 (35.1)
4-year college graduate	1 (4.2)	9 (45.0)	7 (35.0)	2 (20.0)	19 (25.6)
Some school beyond college	1 (4.2)	0 (0.0)	0 (0.0)	0 (0.0)	1 (1.35)
Graduate or professional degree	0 (0)	1 (5.0)	8 (40.0)	7 (70.0)	16 (21.6)
**Income**					
<$25,000	11 (45.8)	0 (0.0)	4 (20.0)	1 (10.0)	16 (21.6)
$25.000–49.000	7 (29.2)	1 (5.0)	11 (55.0)	0 (0.0)	19 (25.6)
$50,000–74,999	4 (16.7)	7 (35.0)	2 (10.0)	2 (20.0)	15 (20.3)
$75,000–99,999	1 (4.2)	6 (30.0)	1 (5.0)	2 (20.0)	10 (13.5)
Over $100,0.000	0 (0.0)	6 (30.0)	0 (0.0)	4 (40.0)	10 (13.5)
Don't know/prefer not to answer role in overdose reporting	1 (4.2)	0 (0.0)	2 (10.0)	1 (10.0)	4 (5.4)
Emergency department/ Hospital employee	–	4 (16.6)	0 (0.0)	2 (20.0)	6 (11.1)
EMS	–	12 (50.0)	0 (0.0)	0 (0.0)	12 (22.2)
Epidemiologist	–	0 (0.0)	0 (0.0)	2 (20.0)	2 (3.7)
Fire department	–	6 (25.0)	0 (0.0)	1 (10.0)	7 (12.9)
Harm reductionist	–	1 (4.1)	18 (90.0)	0 (0.0)	19 (35.1)
Law enforcement officer	–	1 (4.1)	0 (0.0)	0 (0.0)	1 (1.9)
Poison control	–	0 (0.0)	0 (0.0)	1 (10.0)	1 (1.9)
Substance use treatment provider	–	0 (0.0)	2 (10.0)	1 (10.0)	3 (5.6)
Other experts	–	0 (0.0)	0 (0.0)	3 (30.0)	3 (5.6)

### Preservation of Trust

This theme emerged throughout the design process and across all community stakeholders. The harm reduction and PWUD noted that it was imperative that we embrace the mantra: “Nothing about us without us” and develop a tool that is “Informed by the community, for the community.” Harm reduction stakeholders noted how important preserving trust among their clients is toward operational success of the organization. They have worked hard on the ground to develop relationships with community gatekeepers and establish their organization as worthy of trust among the drug using community. As such, any technology developed through this co-design process must put the community first, above the academic and funder's priorities.

Our community advisory boards emphasized the importance of trust in being able to capture data from “hidden populations” who do not come into contact with the healthcare system and are not captured in existing overdose surveillance methods. One harm reduction leader stated: “I would say [non-reported overdoses] are pretty high... If I was being conservative, maybe 50 to 60 percent [of overdoses go unreported]. I'd have to say [current overdose surveillance data] is very inaccurate” (120, Harm Reductionist).

The advisory boards and harm reduction organizations viewed obtaining data from this population as key to be able to have real-time, meaningful data that will inform community overdose prevention and response efforts. “I do think a more comprehensive app and website would be useful, but then like, more trust would have to be established and that there would also have to be ways to…ensure that people without access to technology would, maybe even like, maybe if there is like, an incentive for people to like, report an overdose, but I think that would definitely have to be carried out by a harm reduction organization because I just feel like that's where most trust is placed in the community” (121, PWUD).

### Preferences for Content and Technical Features of the Digital Platform

In regards to technical features, participants commented on the need for ease of use, discussed the complexity of location documentation, and identified several features for the digital platform.

#### Ease of Use

In terms of technological features, participants highlighted a need for flexible data entry methods, offline usage capability, and for simplicity and ease. First responders requested simplicity but also repeatedly noted compliance might be poor and that pulling data from the existing system would be better because they already have systems in place. One first responder stated “don't make something complicated and I think it will piss people off if they are like, ‘I already entered this”' (146, EMS). Harm reduction workers and PWUD commented on both apps and website portals. Participants highlighted the importance of accessibility as it relates to equity in adoption and data reliability: “if different people are going to be using it you have to make it really easy to understand because then you get misclassification of information.” (106, Harm Reductionist). Another participant noted, “It should be very practical and easy to use, uh, where they are not able—where they're able to just, um, like a one-, two-, three-step—not make it more than that because, uh, they might get fatigued, as it is, they're already using [drugs], you know—-so their patience is not too good, you know, so I think we have to keep that app very practical” (141, Harm Reductionist).

The ability to download an app in the iTunes and Google Play stores was often mentioned but responses included those who preferred to log into a web portal because some participants expressed privacy concerns regarding a mobile application: “I think that most people have phones, right? Um, even folks that are homeless out in the community have phones. So I think that, if there was a really easy, free, downloadable app that people could use to report these, I think that they would report “em”” (108, Harm Reductionist). One PWUD felt an app would be fine for their peer group but would not work for everyone because of phone access issues: “For my cohort of people, an app would be very effective. But for people that don't have or use apps, that's not gonna help them” (136, PWUD). Another PWUD commented on concerns about privacy with an app: “Not something that, like, a lot of things that you gotta sign up and put your name and put your email and create a username and a password and all that, like, no” (158, PWUD). This was echoed by other PWUD, “I don't trust the phones” (160, PWUD).

Regarding simplicity and ease of use, one EMS worker said “Yeah, I think you're gonna get a lot more use if it's kind of binary, in the sense that it's—you know, you can just—you can click through options. You know, the less that someone has to freeform an answer, and I hate to say it, but like the less somebody has to write a narrative, the more likely it is that they'll use it consistently because it's easy” (147, Firefighter).

Many participants ranging from harm reduction workers to PWUD highlighted a desire to use the platform quickly and many brought up drop down options. One law enforcement officer highlighted utility of drop downs: “I mean, guess ease of use, right. Drop-down menus are super easy. You just click-click check boxes or whatever” (144, Law Enforcement Officer). For EMS workers, integration with current systems was a highly desired technical feature. One EMS worker felt this was critical to utility: “So, having it integrated into my EPCR and makin' it to where I can't close it without doing it would be the only way to get the 100 percent compliance” (148, EMS).

#### Location Documentation

Preferences on overdose incident location documentation varied between those who valued granularity down to the zip code or community/neighborhood level. Harm reduction workers noted that more precise location data would allow them to make data-driven decisions for community outreach strategy. First responders noted zip codes would be helpful whereas PWUD noted areas of town or general neighborhoods where the overdose occurred, and more importantly to them, where the drugs were purchased. Taken together, there seemed to be perceived value in documentation of location of overdose, but groups viewed locations somewhat differently.

For example, one first responder (143, Firefighter) said “Oh, man. Well, if it's going up to the state, I would think some sort of, you know, uh, well, the state employees should be able to have access to it so that maybe they can see if they're having, like, a spike in, say, zip codes or that kind of thing. You know what I mean? That way they could, like, maybe have better, uh, communication with the local municipalities as far as, you know, “We've been tracking numbers and we show that in your zip code that it spiked, like, 20 percent,” or whatever. You know? That kind of thing...Yeah. I think it should be local and state should have access to it. Definitely.”

One harm reduction worker in an urban area said “I think at least being able to see if there are neighborhood clusters. Or a particular area. I mean that would be incredibly helpful for our services. I mean, if we could see that a bunch of people overdosed even if we didn't know from what, if there is like a spike in the map. And we–and other organizations–could figure out what's going on in that community” (105, Harm Reductionist). Another harm reduction worker highlighted the need to document where the drugs were from, not just where the overdose occurred. “if I'm still using drugs part of what I wanna know is where did the drugs come from. People get drugs from different places, so one of them is like a hotspot for someone who deals drugs that are knowingly filled with fentanyl” (106, Harm Reductionist). This was echoed by PWUD “...and they can also report whether an emergency call was made, whether Narcan was used or not used, what substance it was, just a general part of town” (122, PWUD).

#### Features

Features desired included components such as documentation of overdose context, polysubstance use, interventions administered such as reversals or rescue breathing, and location of the overdose incident. A popular feature among harm reduction workers was the ability to track resources such as naloxone. For example, one harm reduction worker had the idea that this could facilitate resource sharing: “sometimes, um, I ran-I ran out of testing strips or I ran out of Narcan and then, um, usually I just ask other agencies in the area, ‘Hey do you—can I have some testing strips or Narcan?’ And they usually have a bunch that they didn't use. So maybe a button or a link to ordering more” (140, Harm Reductionist). This was echoed by an EMS worker: “Um, I think if-if Narcan was available in the community, that would be helpful to be able to know, like, where you could get it, how you could get it. That sort of thing. And then, if you did have it and you administered it, then it would be nice, like you said, to have an app or some sort of software or internet access to where you could document like, “Yes, I did use this community, uh, resource, and it was effective.” You know what I mean?” (142, Firefighter).

PWUD added additional features related to the desire for the website to include resources such tutorials on vein care or naloxone, or where to get treatment. One PWUD noted, “like some of these, um-some of these, uh, website or, um, mobile apps, um, they have, uh-they have a option on there where you can either, you know, go into like a frequently asked questions section— or you can type in, um, you know, a keyword like “help” or, um, uh, you know, “information” or “info” or something. And then you can have some kind of bot respond back to you, you know, about giving you options about what you, you know, want information on, or what you want help with. You know, and you can get all kinds of resources like that, you know, if - if you're looking to get some kind of, uh, um, recovery services or, um, emergency services, you know, poison control numbers. Yeah. Yeah, anything like that, um, I - I - I think that's definitely something that either already exists or should exist by now, but, um, yeah, that's definitely a—I think it's a good idea.” (122, PWUD).

One harm reduction worker added that including treatment resources may require tailoring to each community: “Here are our services. Here are different organizations,” and that kinda, like, for example—but then the problem with that is you're gonna have to regionalize it. “Cause, like, somebody in, uh, Dallas has no benefit from knowing that [harm reduction organization] does HIV testing from one through five” (138, Harm Reductionist).

### Opportunities and Concerns for the Digital Platform

Participants were provided the opportunity to reflect on how overdose reporting could be improved. Themes from these questions included data accessibility, data integration, and privacy concerns.

#### Data Accessibility

Regarding the technology, harm reductionists and the SUD treatment providers endorsed accessibility of the surveillance data. Some harm reductionists were excited about the idea of unified data across the state, and having “Just one system where everyone could go to one place” (104, Harm Reductionist). One PWUD suggested that alerts could be used to notify users when overdose rates increase in their area (122, PWUD). Several EMS/Fire respondents noted that they already record overdose data, and so an efficient system would pull data from their records: “the system that we have right now is pretty good. I'm happy with it, and, like, it has all that stuff you need as far as drugs and-and overdose tracking” (142, Firefighter). Several EMS/Fire respondents suggested that state-mandated reporting would be helpful: “I think that if the state were to mandate collection of the data, then I could see administration either mandating us to go and fill out those forms and referrals, or just designating somebody to collect that data” (149, EMS).

#### Data Integration

Harm reductionists, EMS/Fire, and poison control respondents highlighted the importance of combining data sources: “there's so many different systems that trying to make sure that it isn't a duplicate or that we're not missing something or, you know, whatever it is, um, that's probably, one of the harder things” (153, Poison Control). Another participant highlighted challenges with obtaining real-time overdose data and the need for data aggregation across systems: “I don't think you can create anything that's gonna give you all the information in real time that is experiencing overdose. I don't. You're gonna need a combination of reporting” (109, Harm Reductionist).

Some harm reductionists suggested that shared data could create opportunities for collaboration across harm reduction organizations, while others suggested broader impacts of reporting for PWUD: “I feel like that should be the goal, to empower our [clients] to be able to report that information when needed and as they feel comfortable” (116, Harm Reductionist). Another harm reductionist highlighted how this might facilitate empowerment among PWUD: “That would empower them, that would make them feel like they are worthy of being cared for and better” (110, Harm Reductionist). Another harm reductionist noted, “The idea is empowering people in their own health because there is a lot of things in their life that they can't control” (106, Harm Reductionist). Similarly, one PWUD suggested that more reporting data might help people realize how much more common overdoses are, and this would allow for increased funding (136, PWUD).

When asked about their open-ended goals, harm reductionists commonly cited the need for changes to public policies in Texas: “I think it would be great if there was buy-in from all of our counties across the state…. I think you could create an amazing infrastructure and website, and then if you don't have the political will to get people to use it, then it will be similar to some of the stuff we already have [i.e., non-integrated platforms such as OD Map and TONI]” (105, Harm Reductionist). Another participant noted, “I would first have to change state policy where programs have to collect this information or are allowed to work with this population without fear of losing their funding. So I would include state policy to include harm reduction services” (107, Harm Reductionist).

#### Privacy

PWUD were very concerned with anonymity. Their primary concern was ensuring that law enforcement cannot access their personal information. “People don't report it because they are afraid. Because of an underlying mistrust” (136, PWUD). This mistrust was reflected in their suggestions for policy-related improvements. PWUD indicated that their peers were unlikely to report overdoses through existing channels due to mistrust, and enhancing trust would be an effective path to improved reporting: “If people started having more positive experiences when they did report it, then, you know, word would get around what really happens when you report it” (135, PWUD). This same participant then noted: “Like, that's how it happens when you report an overdose. Like, they don't frisk everybody there and threaten to throw them in jail” (135, PWUD). Notably, EMS/Fire respondents recognized this perception, but felt it was not accurate: “The perception is that if law enforcement's gonna get involved, you know, I'm gonna go to jail, or my friend's gonna go to jail. And [we need] some kind of massive public education campaign that explains to people that, you know, hey, this is not a criminal activity” (147, Firefighter).

## Discussion

Emerging research estimates that 50–70% of overdoses in Texas go uncounted as a result of the punitive and stigmatizing nature of policies and the fact that only 15 of the 254 counties in Texas have a medical examiner to diagnose overdose as a cause of death (16). Many PWUD experiencing or witnessing an overdose do not contact EMS nor healthcare providers due to stigma and fear of legal repercussions. This fear is well-founded. Texas both lacks a Good Samaritan Law and has more punitive drug use policies (17, 18), with the result that many people who use drugs (PWUD) are swept into the criminal justice system. Further, Black adults are more than twice as likely to be arrested for drug possession and nearly four times more likely to be arrested for marijuana possession relative to White adults exacerbating racial disparities (19–21). Accurate overdose surveillance data is needed to facilitate system and policy change. Capturing data from PWUD and the harm reduction community is necessary to better understand the overdose crisis in Texas and improve reliability of data.

This study highlighted the importance of using a co-design process in the development of an overdose surveillance digital platform to facilitate equity across multisectoral partners including harm reduction workers, first responders, and people who use drugs. Community engagement throughout the development process is critical toward developing digital health tools for underserved people who use drugs. We combined community-engaged research and user-centered design methods to serve as the foundation of the co-design process with our community, academic, and industry partners (8, 10, 11). Dismantling the power structure among academic and industry partners was a critical initial step toward creating equity in engagement of community-based partners, particularly among persons with lived experience in addiction, a history of incarceration, or financial challenges. This was accomplished through community advisory boards, qualitative interviews, and hiring paid consultants which included three local street artists with lived experience, and a first responder and harm reductionist. Results from this study highlighted several key components in developing a community-driven overdose reporting platform. First, preserving trust between harm reduction organizations and their clients is critical. This warrants a digital platform that is safe and secure, and protects their clients from potential legal repercussions. As such, PWUD want an option for anonymous reporting. Accessibility considerations should take into account community members who do not have or use mobile devices or speak fluent English. Further, reporting should be easy, quick, and simple. Less required data points may increase the number of reports. Incorporating flexible data entry methods may facilitate adoption, such as incorporating a speech-to-text feature, data capture through taking a picture of a written report on paper, having a call-in hotline, and pulling data from the backend of existing systems.

**Figure 2:**
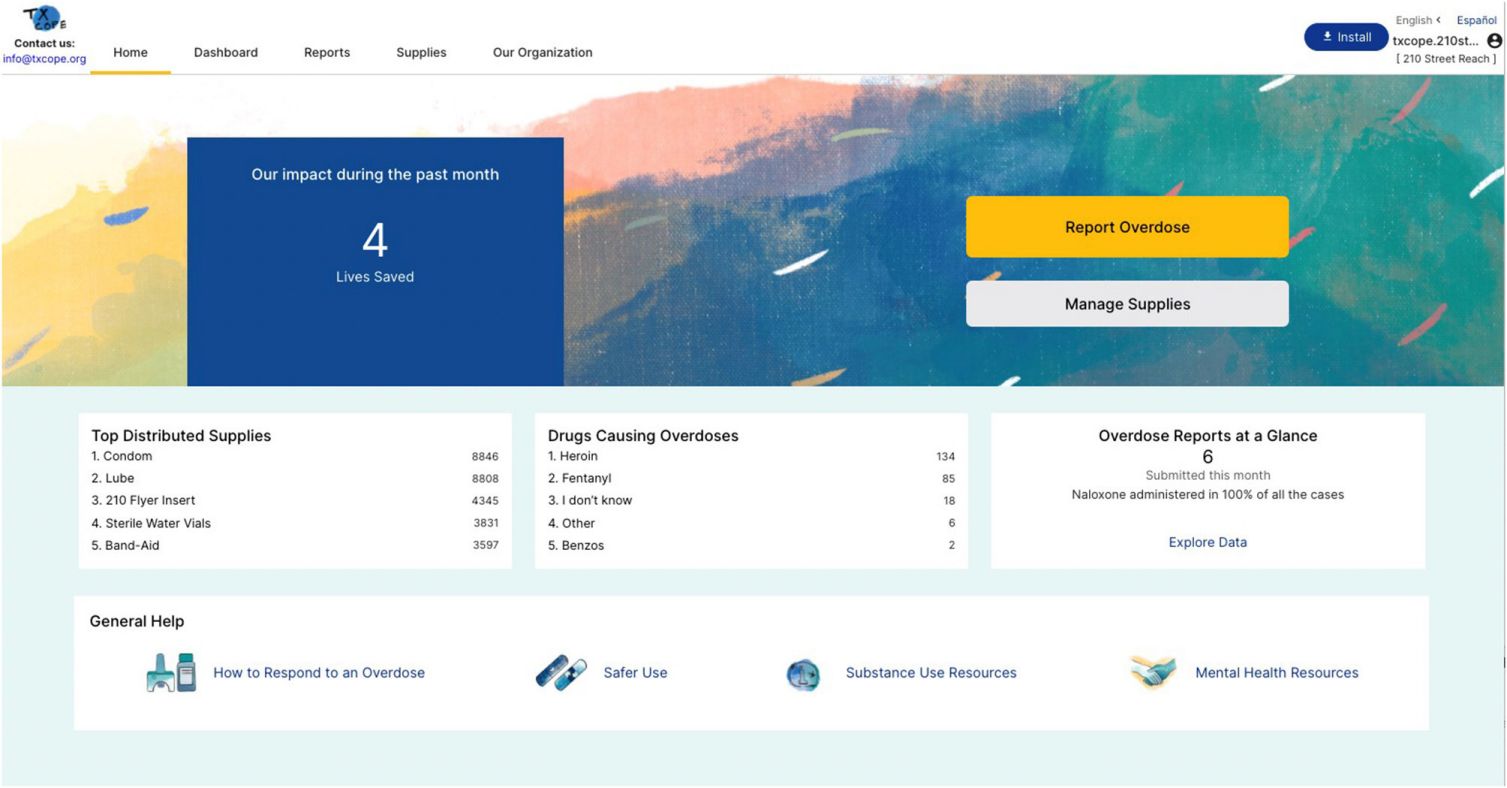
TxCOPE landing page design for harm reduction organizations.

**Figure 3:**
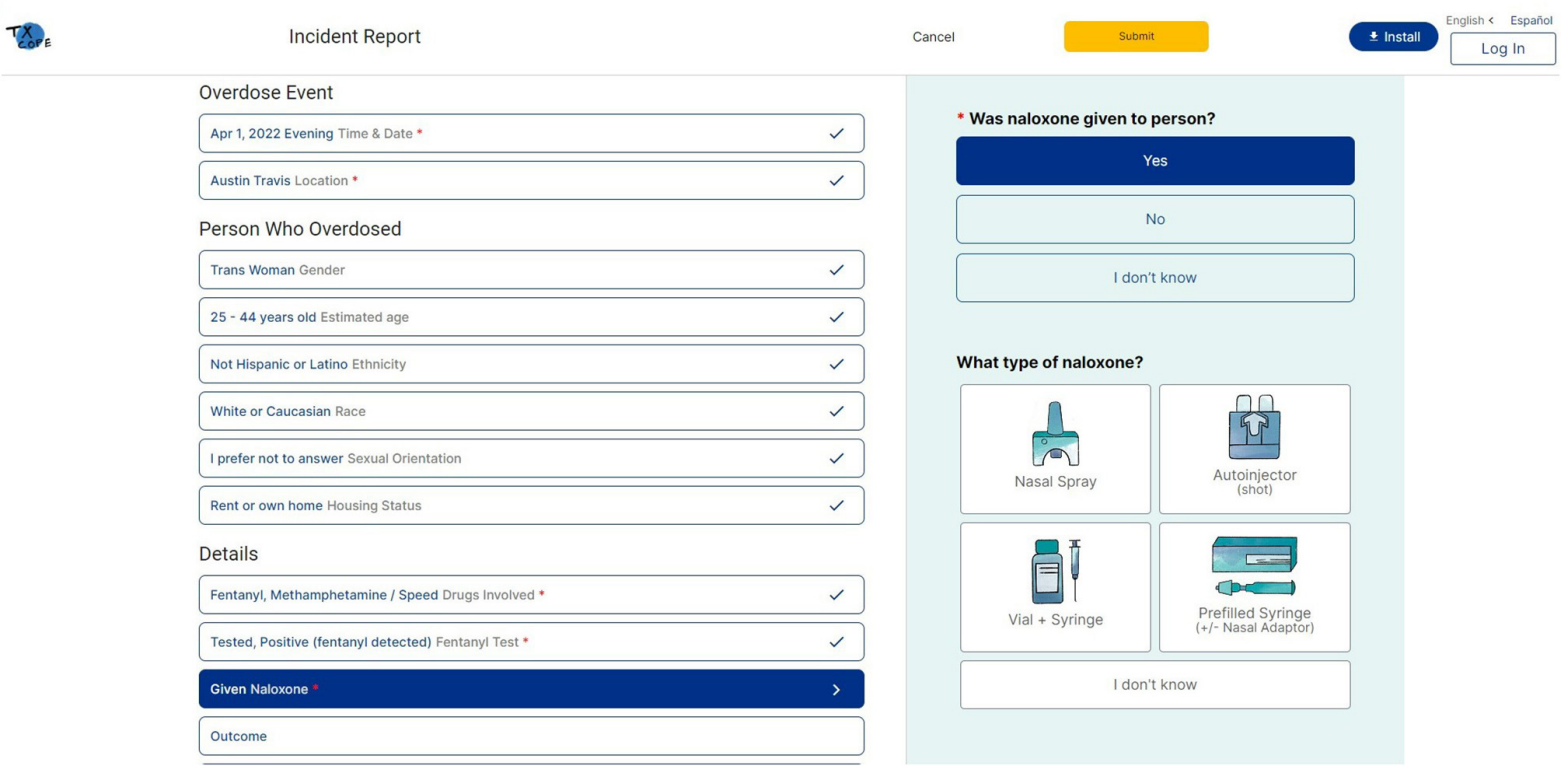
TxCOPE overdose incident report form.

Our co-design process resulted in the development of the TxCOPE dashboard uniquely tailored to harm reduction organizations (see Figure 2) (www.txcope.org/harmreduction). Priorities of the community included HIPAA-compliant, secure, and anonymous reporting form. The overdose report form was designed by the community advisory board (CAB) members with detailed attention to language used throughout the report form. The community wanted to ensure data will be collected for marginalized populations with a priority among trans-people and racial/ethnic populations (see Figure 3). The CABs also designed a supply tracking portal that enables harm reduction organizations to manage supply distribution and map locations where supplies are given during community outreach. This allows organizations to have data to drive their supply distribution efforts and see if their community outreach efforts maps on to the same locations where overdoses are occurring. Finally, the CABs designed the data dashboard ensuring data is displayed at the organization level, county level, and state level. The data dashboard was designed to facilitate harm reduction organizations ability to demonstrate their community impact and easily insert graphs and figures from the dashboard into grant applications.

Findings from this study should be taken in light of several limitations. First this study was exploratory in nature and only used qualitative methods. As such, we cannot generalize these findings of key stakeholders beyond the state of Texas. Participant perceptions likely reflect the policy infrastructure existing in Texas at the time of this study. Our sample included first responders; however, law enforcement was underrepresented in our sample. Future studies should seek to better understand perspectives on overdose reporting and use of data among law enforcement and criminal justice stakeholders.

This study highlighted a multisectoral co-design process across community-academic-industry partners to develop a digital health tool tailored to the unique needs of community-based harm reduction organizations serving highly vulnerable people who use drugs. These partnerships are critical toward creating impact and reducing health disparities among highly vulnerable people who use drugs. Incorporating non-traditional first responders into overdose surveillance methods is essential toward capturing data among hidden populations. This is a needed first step in promoting equity of overdose prevention and community outreach among highly vulnerable people who use drugs.

## Data Availability Statement

The raw data supporting the conclusions of this article will be made available by the authors, without undue reservation.

## Ethics Statement

The studies involving human participants were reviewed and approved by The University of Texas at Austin Institutional Review Board. The patients/participants provided their written informed consent to participate in this study.

## Author Contributions

KC is the principal investigator for the study, led the conceptualization of the study, and the drafting of the original grant proposal. SC and JB are co-investigators and contributed to conceptualization of the study, contributed to writing of the original grant proposal, and successive drafts of the manuscript. QW, KC, SC, and JB developed a first draft of the manuscript. QW assisted with data collection, data analysis, and drafting the manuscript. All authors read and approved the final manuscript.

## Funding

This study was supported by Texas Targeted Opioid Response, a public health initiative operated by the Texas Health and Human Services Commission through federal funding from the Substance Abuse and Mental Health Services Administration (SAMHSA) grant award number 1H79TI081729. KC effort was supported, in part, by NIDA K23DA039037. The funder had no role in the design and conduct of the study; collection, management, analysis, and interpretation of the data; or preparation of the manuscript.

## Author Disclaimer

The content of this study does not represent the official view of SAMHSA, NIDA, or the Texas Health and Human Services Commission.

## Conflict of Interest

The authors declare that the research was conducted in the absence of any commercial or financial relationships that could be construed as a potential conflict of interest.

## Publisher's Note

All claims expressed in this article are solely those of the authors and do not necessarily represent those of their affiliated organizations, or those of the publisher, the editors and the reviewers. Any product that may be evaluated in this article, or claim that may be made by its manufacturer, is not guaranteed or endorsed by the publisher.
